# New species of *Trophoniella* from Shimoda, Japan (Annelida, Flabelligeridae)

**DOI:** 10.3897/zookeys.614.8346

**Published:** 2016-09-01

**Authors:** Naoto Jimi, Yoshihiro Fujiwara

**Affiliations:** 1 Graduate School of Biosphere Science, Hiroshima University, Higashi-Hiroshima 739-8528, Japan; 2 Department of Marine Biodiversity Research, Japan Agency for Marine-Earth Science and Technology, Yokosuka 237-0061, Japan; 3 Department of Natural History Sciences, Graduate School of Science, Hokkaido University, N10 W8, Sapporo 0060-0810, Japan

**Keywords:** Nabeta Bay, Polychaeta, tank, taxonomy

## Abstract

*Trophoniella hephaistos* **sp. n.** was collected from a tank irrigated with seawater pumped directly from Nabeta Bay, Japan. This species is discriminated from other *Trophoniella* by having dorsal tubercles, a tongue-shaped branchial plate, a tunic covered with large sediment grains dorsally and ventrally, having eyes, and anchylosed neurohooks starting from chaetigers 17–20. This is the first record of *Trophoniella* from Japanese waters. Identification keys to species of *Trophoniella* and four gene sequences (*COI*, *16S*, *18S*, *28S*) of this species are provided. Phylogenetic analysis was conducted to clarify phylogenetic position of *Trophoniella* in Flabelligeridae using four genes.

## Introduction

*Trophoniella* Hartman, 1959 belongs to the family Flabelligeridae and currently consists of 25 species and one undescribed species (Salazar-Vallejo 2012b). *Trophoniella* polychaetes live in sediments from shallow water to the deep sea in tropical or subtropical regions (Salazar-Vallejo 2012b). This genus is characterized by having anchylosed neurohooks in the median or posterior chaetigers, bidentate or bifid tips, a thick tunic, a tongue-shaped branchial lobe (except for *Trophoniella enigmatica*), and longitudinal rows of elongated single papillae along the body (Salazar-Vallejo 2012b). *Trophoniella* resembles *Piromis* and *Pycnoderma* in having a thick tunic, often with sediment grains, a tongue-shaped branchial lobe, and multiarticulated notochaetae. However, it is distinct from *Piromis* and *Pycnoderma* by having anchylosed neurohooks in the median or posterior chaetigers (Salazar-Vallejo 2011b).

Nine flabelligerid genera have been recorded from Japanese waters to date, i.e., *Brada*, *Buskiella*, *Daylithos*, *Diplocirrus*, *Flabelligera*, *Pherusa*, *Piromis*, *Semiodera*, and *Stylarioides* (Imajima 1964; Imajima 2006; 2009; Imajima and Hartman 1964; Miura 2014; Salazar-Vallejo 2011a; Salazar-Vallejo 2011b; 2012a, b; 2014; Salazar-Vallejo and Buzhinskaja 2011; Uchida 1992). However, *Trophoniella* was not recorded from Japan in previous studies.

Phylogenetic analyses of Flabelligeridae were conducted several times by using morphological and molecular data sets (Burnette et al. 2005; Osborn and Rouse 2008; 2011; Salazar-Vallejo et al. 2008). A morphological analysis suggested that *Trophoniella* was similar to *Piromis*. However, the molecular data was unable to robustly resolve the phylogenetic position of *Trophoniella*; this is likely an artefact of limited taxon sampling within the genus.

During benthos sampling in an aquarium in the Shimoda Marine Research Center (SMRC), University of Tsukuba, we collected undescribed species of *Trophoniella*. Here, we describe *Trophoniella hephaistos* sp. n. and cytochrome *c* oxidase subunit I (COXI), 16S ribosomal RNA (16S), 18S ribosomal RNA (18S), 28S ribosomal RNA (28S) gene sequences to contribute to the DNA barcoding of the Flabelligeridae. A phylogenetic analysis was conducted using four genes to clarify relationships of *Trophoniella* within the family Flabelligeridae. To the best of our knowledge, this is the first report of *Trophoniella* from Japanese waters.

## Material and methods

**Figure 1.**
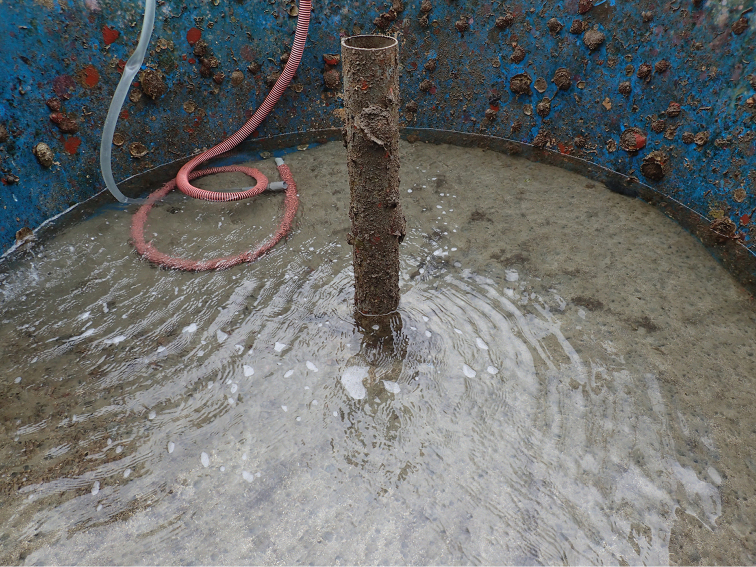
Sampling location of *Trophoniella hephaistos*. Worms were collected from a tank continuously irrigated with seawater pumped directly from Nabeta Bay at a depth of 3 m.

Worms were collected by hand from a tank (MF-5000S, aquaculture system, Japan. 2.4 m in diameter and 1.1 m in depth) installed in the SMRC, University of Tsukuba, Shizuoka (34°40.045'N; 138°56.145'E) (Fig. 1). The tank contained sandy mud and sea water and the worms lived between 0 and 30 cm below the sediment surface. Seawater in the tank was drawn only from Nabeta Bay, directly in front of the SMRC, from a depth of 3 m (location of the head gate: 34°39.950'N; 138°56.283'E). Several samplings were conducted in Nabeta Bay and other surrounding sites at depths between 2 and 386 m by the first author and members of the SMRC but there was no individual of *Trophoniella* discovered except in the tank. All the specimens were first anesthetized with menthol and then fixed and preserved in 70% ethanol. The anesthesia duration differed among samples. Preserved specimens were observed under stereoscopic MZ 16F (Leica, Germany) and E600 (Nikon, Japan) microscopes. All specimens were deposited in the National Museum of Nature and Science, Tokyo (NSMT), Japan.

Genomic DNA was extracted from a small piece of the epidermal tissue of the holotype (NSMT-Pol-H-601) using the DNeasy Blood & Tissue Kit (Qiagen, USA) following the manufacturer’s protocol. Partial cytochrome *c* oxidase subunit I (*COXI*), 16S ribosomal RNA (*16S*), 18S ribosomal RNA *(18S)*, 28S ribosomal RNA (*28S*) gene sequences were amplified in the polymerase chain reaction (PCR) with the primer sets of polyLCO (5’-GAYTATWTTCAACAAATCATAAAGATATTGG-3’) and polyHCO (5’-TAMACTTCWGGGTGACCAAARAATCA-3’) (Carr et al. 2011), 16SarL (CGCCGTTTATCAAAAACAT) and 16SbrH (CCGGTCTGAACTCAGATCACGT) (Palumbi et al. 1991), mitchA (CAACCTGGTTGATCCTGCCAGT) and mitchB (TGATCCTTCCGCAGGTTCACCTAC) (Medlin et al. 1988), and LsudiF (ACCCGCTGAATTTAAGCATA) and D3aR (ACGAACGATTTGCACGTCAG) (Lenaers et al. 1989) , respectively. The reaction mixture [0.25 µl TaKaRa Ex Taq (Takara, Japan), 5 µl of 10 × Ex Taq Buffer (Takara, Japan), 4.0 µl dNTP mixture (Takara, Japan), 5 µl of each primer pair (10 µM), 0.75 µl of extracted DNA, and 35 µl of distilled water] was used for amplification. The PCR protocol for *COX1* consisted of an initial denaturation step at 94 °C for 1 min, followed by 35 cycles of 30-s denaturation at 94 °C, 60-s annealing at 50 °C, and 1-min extension at 72 °C, and a final extension at 72 °C for 10 min. The PCR protocols for *16S*, *18S*, *28S* were followed an previous study (Osborn and Rouse 2011). To confirm successful amplification, PCR products were visualized using 1.2 % Agarose S (Nippon Gene, Japan) gel electrophoresis. The DNA sequencing reaction of the PCR products was performed using the BigDye Terminator v3.1 Cycle Sequencing Kit (Applied Biosystems, USA). Direct sequencing was performed using the 3130xl Genetic Analyzer (Applied Biosystems, USA). Sequencing reactions were conducted using the 1-µM primers applied for the PCR amplification. The newly obtained sequences were deposited in the DNA Data Bank of Japan (DDBJ) (accession nos. LC136932, LC152760, LC152761, and LC152762).

Additional sequences of Flabelligeridae, Acrocirridae, Cirratulidae were obtained from GenBank (following Osborn and Rouse (2011)) (Table 1). All sequences were aligned using Mafft ver. 7.205 under the E-INS-i strategy (Katoh and Standley 2013). Alignment-ambiguous positions were removed using trimAL under the gappy strategy (Capella-Gutiérrez et al. 2009). Kakusan recommended a GTR+G evolutionary model for each of the genes (Tanabe 2007), a phylogenetic tree was constructed using maximum likelihood (ML) methods in the program RAxML-VI-HPC (Stamatakis 2006). The robustness of the ML tree was evaluated by 1,000 bootstrap replicates (-f option).

**Table 1.** List of flabelligerid, acrocirrid, and cirratulid species included in the phylogenetic analysis, together with accession numbers in GenBank.

Taxon	*18S*	*28S*	*COI*	*16S*	Collection site	Reference
**Flabelligeridae**						
*Brada villosa*	EU791460	EU791462	–	HQ326962	Vattenholmen, Sweden	Osborn and Rouse (2008)
*Brada* sp.	HQ326967	HQ326968	HQ326970	HQ326963	Central California, USA	Osborn and Rouse (2011)
*Buskiella* sp.	EU694116	EU694110	EU694128	EU694110	Monterey, California, USA	Osborn and Rouse (2008)
*Diplocirrus glaucus*	AY708534	DQ790031	–	–	Gullmarsfjorden, Sweden	Struck et al. (2007)
*Flabegraviera mundata*	HQ326964	–	HQ326969	HQ326958	South Orkney Islands, Antarctica	Osborn and Rouse (2011)
*Flabelliderma ockeri*	EU694119	–	EU694127	EU694111	La Jolla, California, USA	Osborn and Rouse (2008)
*Flabelligera affinis*	–	DQ779688	–	DQ779614	Iceland	Rousset et al. (2007)
*Flabelligera infundibularis*	EU694118	–	EU694131	EU694112	Astoria, Oregon, USA	Osborn and Rouse (2008)
*Flabesymbios commensalis*	HQ326965	–	–	HQ326959	Malibu, California, USA	Osborn and Rouse (2011)
*Pherusa plumosa*	AY708528	DQ790056	–	–	Central California, USA	Struck et al. (2007)
*Piromis* sp.	–	–	–	HQ326961	Santa Monica, California, USA	Osborn and Rouse (2011)
*Poeobius meseres*	EU694115	EU694123	EU694130	–	Monterey, California, USA	Osborn and Rouse (2011)
*Stylarioides* sp.	HQ326966	–	HQ326971	HQ326960	Spencer Gulf, South Australia	Osborn and Rouse (2011)
*Therochaeta* sp.	AY708527	–	–	–	Woods Hole, Massachusetts, USA	Burnette et al. (2005)
*Trophoniella hephaistos*	LC152761	LC152762	LC136932	LC152760	Shimoda, Shizuoka, Japan	This study
**Acrocirridae**						
*Flabelligena* sp.	EU694120	EU694121	EU694126	EU694113	Pacific Antarctic Ridge	Osborn and Rouse (2008)
*Swima bombiviridis*	GQ422143	GQ422144	FJ944527	FJ944506	Monterey, California	Osborn et al. (2009)
**Cirratulidae**						
*Cirratulus cirratus*	DQ779645	DQ779683	–	DQ779609	Iceland	Osborn et al. (2007)
*Ctenodrilus serratus*	AY340426	AY340388	–	–	Massachusetts, USA	Rousset et al. (2007)

## Results

### Systematics Family Flabelligeridae de Saint-Joseph, 1894 Genus *Trophoniella* Hartman, 1959

New Japanese name: Yoroi-habouki-zoku

#### Trophoniella hephaistos sp. n.

Taxon classificationAnimaliaTerebellidaFlabelligeridae

http://zoobank.org/5A3B2B5C-655E-41CF-B877-31FDFA955E84

New Japanese name: Shimoda-yoroi-habouki

Figs 2 , 3 , 4 , 5

##### Material examined.

Holotype. No. NSMT-Pol-H-601 Incomplete, posterior end absent. Unknown sex, non-reproductive adult, body length 9.0 cm, body width 0.3 cm, 103 chaetigers, 24 September 2015, collected by N. Jimi, tank of the SMRC, sandy mud.

Paratypes. No. NSMT-Pol-P-602. Complete, two specimens. Unknown sex, non-reproductive adult, body length 10.2–11.2 cm, body width 0.4–0.5 cm, 129–141 chaetigers, 24 September 2015, collected by N. Jimi, tank of the SMRC, sandy mud. No. NSMT-Pol-P-603. Incomplete, posterior body absent, nine specimens. Unknown sex, non-reproductive adult, body width 0.4–0.5 cm, 24 September 2015, collected by N. Jimi, tank of the SMRC, sandy mud. No. NSMT-Pol-P-604. Incomplete, posterior body absent, one specimen. Unknown sex, body width 0.3 cm, 26 November 2014, collected by N. Jimi, tank of the SMRC, sandy mud.

##### Diagnosis.

Body covered by large sediment grains dorsally, ventrally, and laterally, without posterior region. Sediment grains not immersed in the tunic. Papillae arise in four rows ventrally and two rows dorsally from first chaetiger to posterior end, longitudinal rows. Tongue-shaped branchial plate. Paired black eyes on center of prostomium. Anchylosed bidentate neurohooks start from chaetiger 17–20, accessory tooth length same as fang.

##### Description.

**Figure 2.**
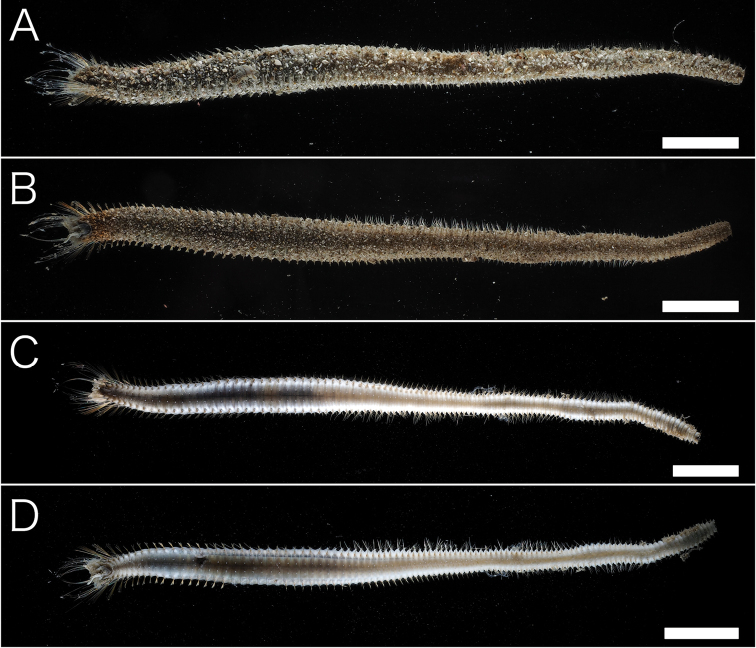
*Trophoniella hephaistos* (holotype: No. NSMT-Pol-H-601). **A** Dorsal view **B** ventral view **C** dorsal view without sediment particles **D** ventral view without sediment particles. Scale bar: 1 cm.

**Figure 3.**
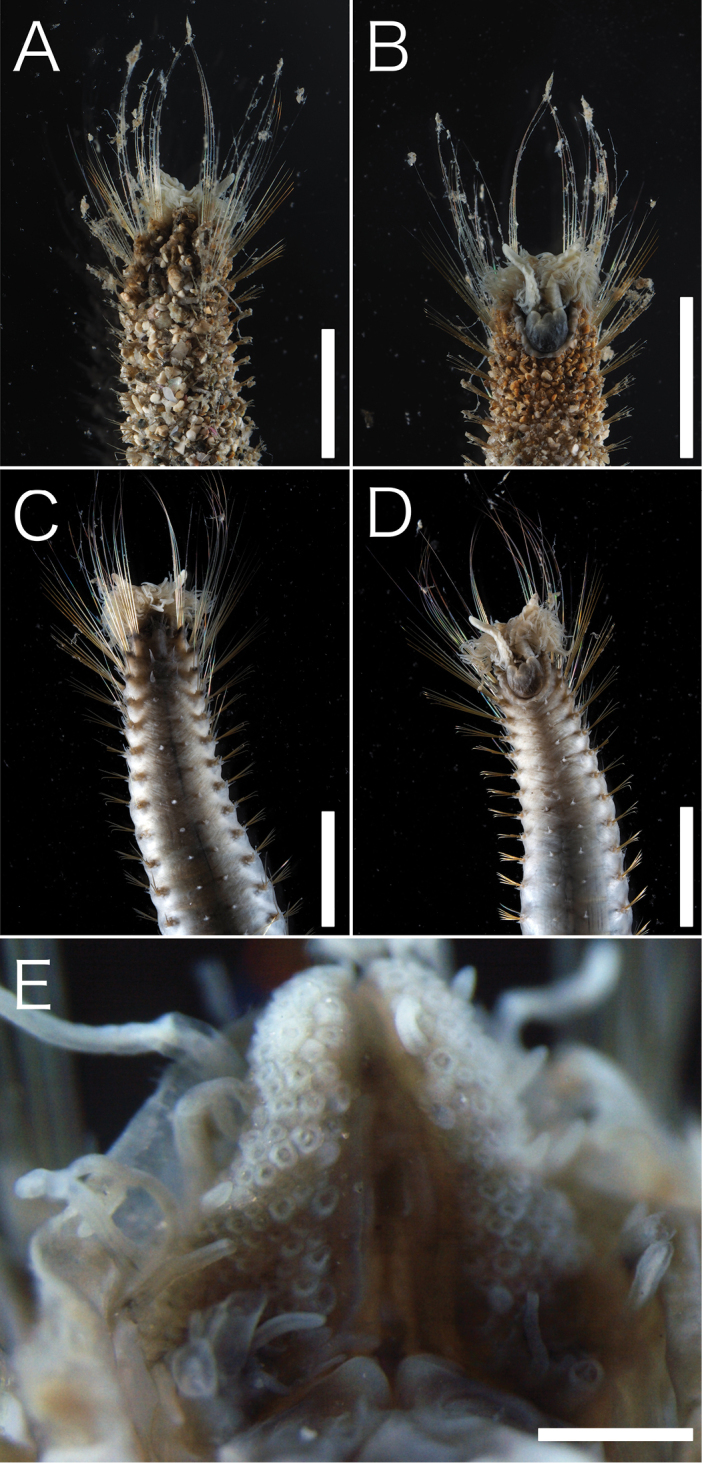
*Trophoniella hephaistos* (holotype: No. NSMT-Pol-H-601). **A** Anterior dorsal view **B** anterior ventral view **C** anterior dorsal view without sediment particles **D** anterior ventral view without sediment particles **E** branchial plate without branchiae and palps. Scale bar: 5 mm (**A, B, C, D**); 0.5 mm (**E**).

**Figure 4.**
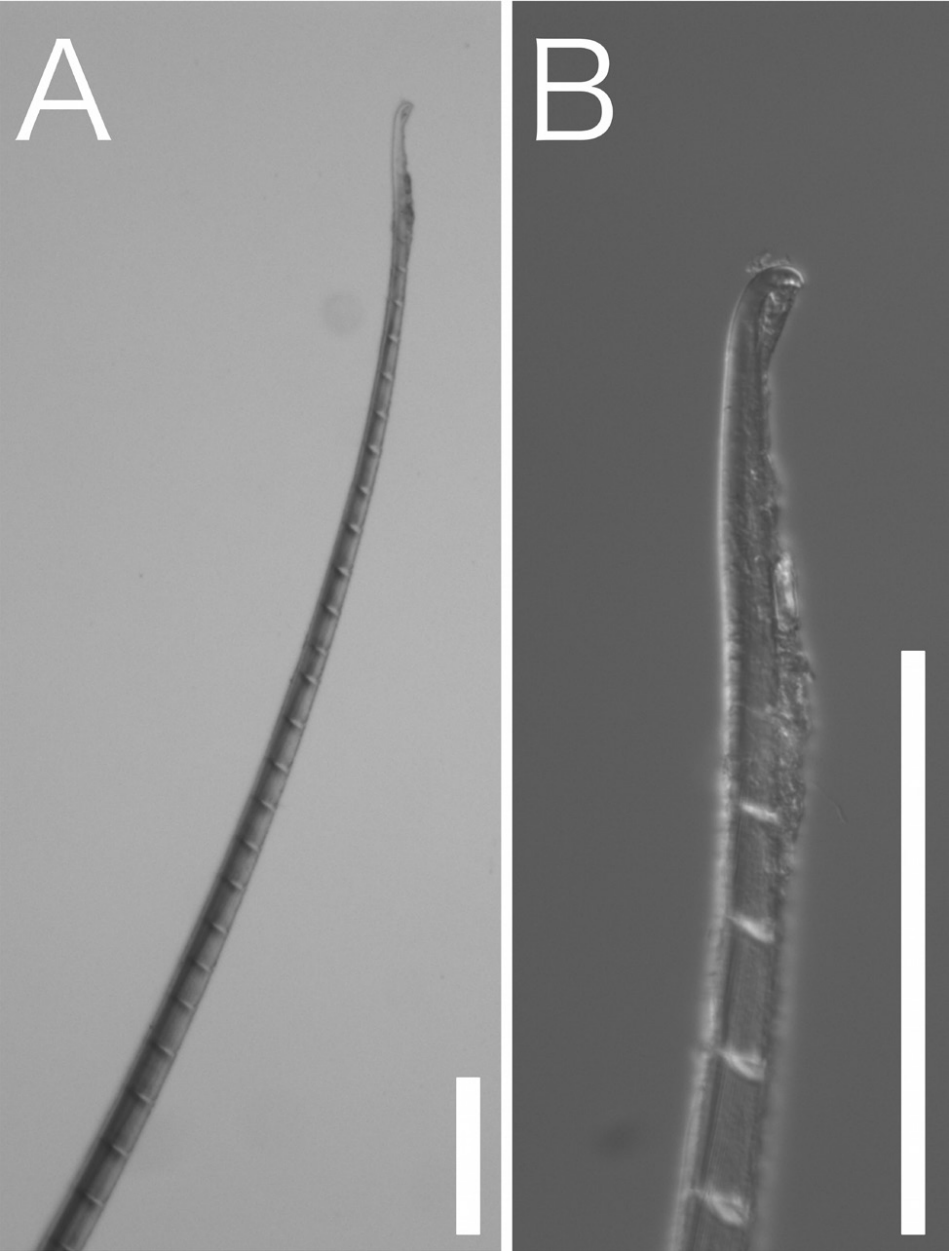
*Trophoniella hephaistos* (holotype: No. NSMT-Pol-H-601). Stereoscopic micrographs of **A** chaetiger 35, notochaeta **B** tip of (**A**). Scale bar: 100 µm.

**Figure 5.**
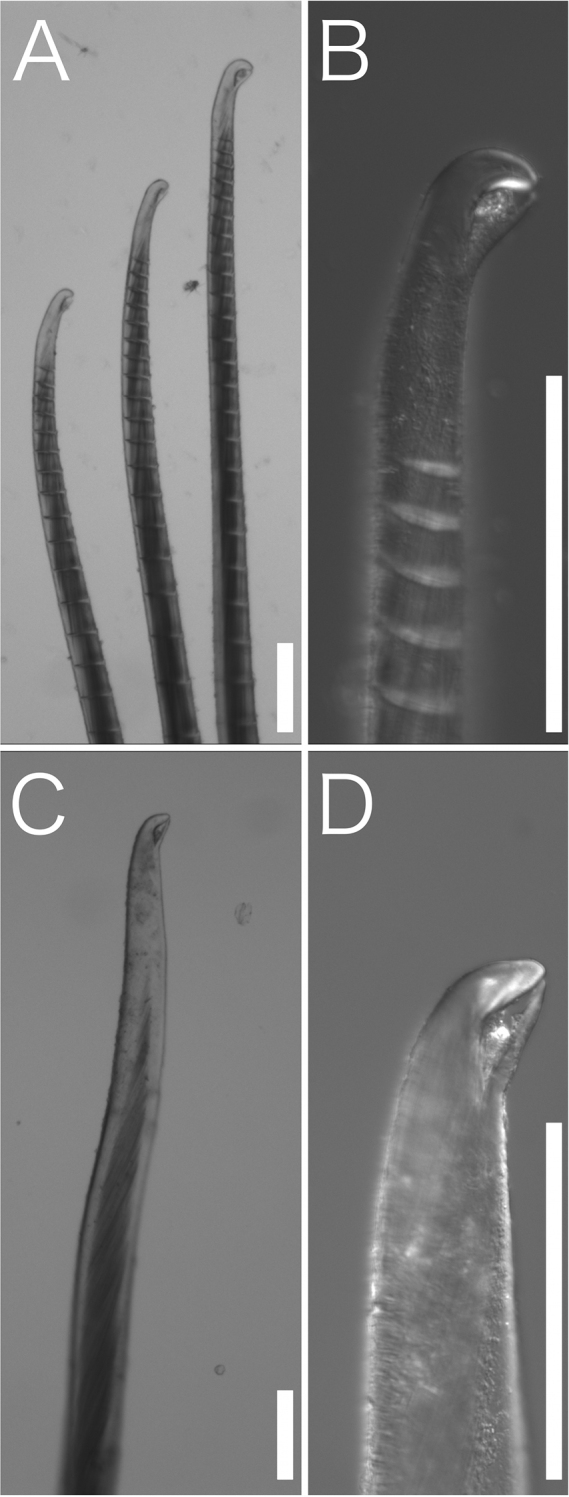
*Trophoniella hephaistos* (holotype: No. NSMT-Pol-H-601). Stereoscopic micrographs of **A** chaetiger 16, neurochaeta **B** tip of (**A**) **C** chaetiger 35, neurochaeta **D** tip of (**C**). Scale bar: 100 µm.

Body length 10.2–11.2 cm (complete specimens), width 0.3–0.7 cm, 129–141 chaetiger (complete specimens). Body white in ethanol, cylindrical anteriorly and tapering posteriorly (Fig. 2). Tunic thick, papillated, with large sediment grains dorsally, ventrally, and laterally (Figs 2A, B, 3A, B), without posterior end region. Sediment grains with long axes of 70–1000 µm, contain sand and shell fragments, not immersed in the tunic. Papillae capitate, sparse, arise in four rows ventrally and two rows dorsally from first chaetiger to posterior end, longitudinal rows. Dorsal 1–6 and ventral 1–3 chaetiger’s papillae are large. Cephalic cage chaetae approx. 1.5 times longer than body width. Chaetiger 1–5 involved in cephalic cage, chaetiger 1 dorsolateral, and chaetiger 2–3 lateral. Chaetal transition from cephalic cage to body chaetae gradual. Chaetiger 1 has about 9 notochaetae and 7 neurochaetae. Anterior dorsal margin of first chaetiger arise multifid lobe (Fig. 3C). Cephalic hood margin papillated, thin, transparent. Caruncle well developed, reaching the end of the tongue-shape branchial plate. Branchia arise from tongue-shaped branchial plate (Fig. 3E), thin, long (0.5–2 mm), green in live, white in ethanol, over 100 filaments arise from two groups (Fig. 3B, D). One pair palps, green in alive, white in ethanol, cylindrical, grooved, long (2 mm in length) (Fig. 3B, D). Prostomium low-cone, paired black eyes on center. Notochaeta all multiarticulated capillaries with articles, bidentate (Fig. 4A, B). Multiarticulated capillary neurochaeta in chaetiger 1, chaetiger 2–16 bidentate neurohooks (Fig. 5A, B). Anchylosed bidentate neurohooks start from chaetiger 17–20 (Fig. 5C, D), yellow, bidentate. Accessory tooth thin, length same as fang. Parapodia poorly developed, chaetae arise from body wall. Noto- and neuropodia have two prechaetal papillae and three postchaetal papillae. Gonopodial lobe absent. Pygidium simple, no anal cirri.

##### Etymology.

The worm is coated with sediment particles, resembling armor. Hephaistos (Ἥφαιστος) was the name of the ancient Greek god of blacksmiths who forged the armor worn by Achilleus. Hephaistos is also spelled Hephaestus. The Japanese name is derived from the type locality (Shimoda), Japanese armor (Yoroi), and flabelligerids in Japanese (Habouki).

##### Distribution.

This new species is currently only known from the tank of the type locality. The seawater in the tank was drawn only from Nabeta Bay from a depth of 3 m directly facing the SMRC. The natural habitat of this species remains unknown. Due to the location of the head gate, *Trophoniella hephaistos* could be a shallow-water species. However, several sublittoral (~50–60 m) invertebrates were collected from this tank (Dr. Hiroaki Nakano, pers. comm.). Additional sampling efforts in Nabeta Bay will clarify the natural habitat of this species.

##### Phylogenetic analysis.

**Figure 6.**
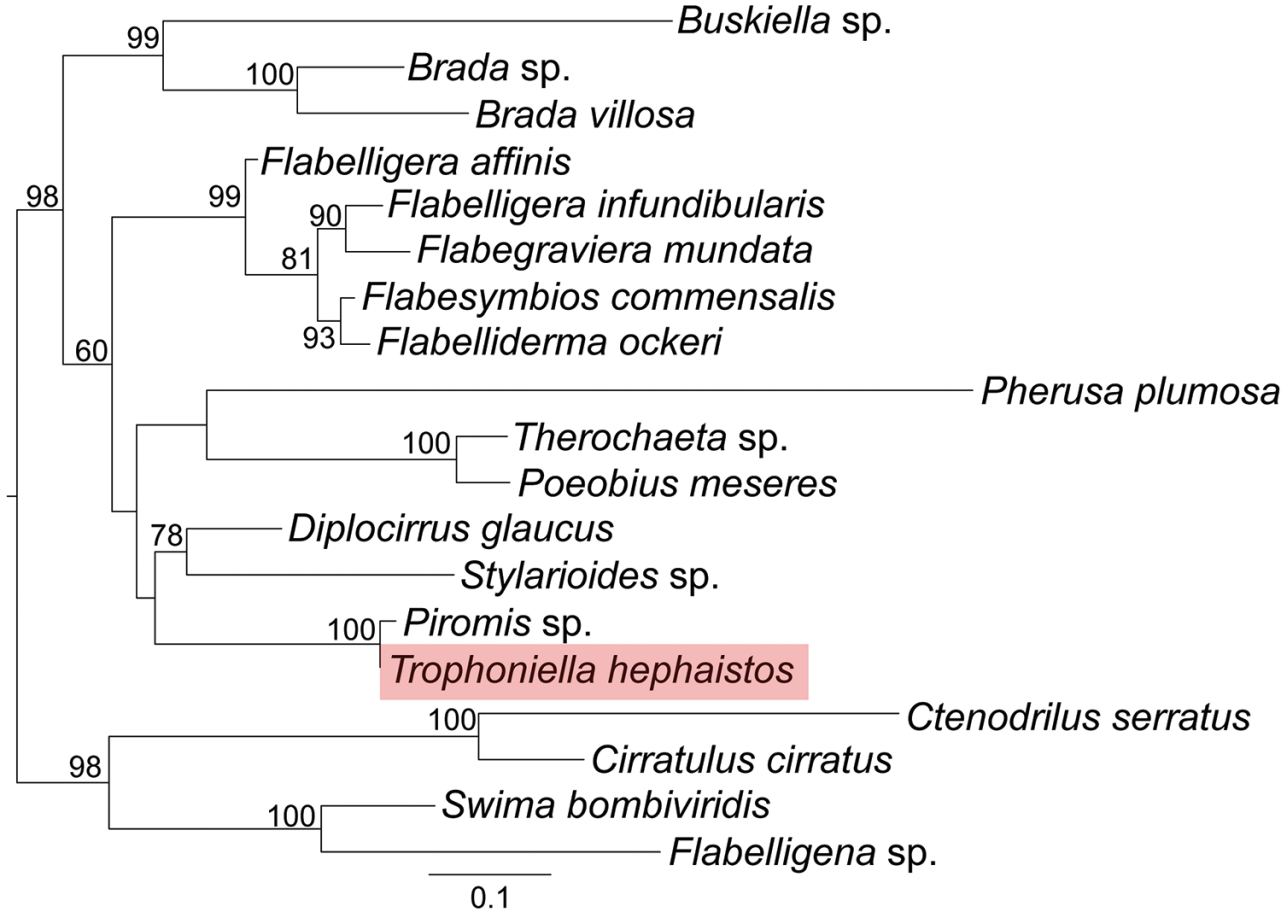
Maximum-likelihood (ML) phylogenetic tree of Flabelligeridae based on *COXI*, *16S*, *18S*, *28S* sequences. *Ctenodrilus serratus*, *Cirratulus cirratus*, *Swima bombiviridis*, *Flabelligena* sp. were used as an outgroup. Nodal support values (bootstrap support value) higher than 50% are indicated on each branch.

The final lengths of the aligned sequences were 669 bp (*COXI*), 485 bp (*16S*), 1893 bp (*18S*), and 910 bp (*28S*). The bootstrap value of 98% in ML analysis strongly supported the monophyly of Flabelligeridae, but internal relationships of Flabelligeridae were not resolved (Fig. 6). The sister group of *Trophoniella* was *Piromis*. The bootstrap value in ML analysis (100%) demonstrated the monophyly of this clade (Fig. 6).

##### Remarks.

*Trophoniella hephaistos* sp. n. resembles *Trophoniella enigmatica* Salazar-Vallejo, 2012 and *Trophoniella indica* (Fauvel, 1928) in having dorsal tubercles at the anterior chaetigers, a tunic covered with large sediment grains dorsally and ventrally, and anchylosed neurohooks starting from chaetiger 14 or posterior. However, *Trophoniella hephaistos* is discriminated by the presence of anchylosed neurohooks starting from chaetigers 17–20, whereas those of *Trophoniella enigmatica* start from chaetiger 40, and of *Trophoniella indica* from chaetiger 14. Additionally, *Trophoniella enigmatica* does not have a tongue-shaped branchial plate and *Trophoniella indica* does not have eyes. Chaetiger number of *Trophoniella hephaistos* was more than twice as many as that of *Trophoniella indica*. *Trophoniella hephaistos* has dorsal body papillae in two longitudinal rows, whereas *Trophoniella enigmatica* in three and *Trophoniella indica* in five.

*Trophoniella hephaistos* also resembles *Trophoniella avicularia* Caullery, 1944 and *Trophoniella harrisae* Salazar-Vallejo, 2012 in having anchylosed neurohooks starting from chaetigers 18–20. *Trophoniella hephaistos* also has dorsal tubercles in the anterior chaetigers, while *Trophoniella avicularia* does not. *Trophoniella harrisae* has sediment particles only on its dorsal area, whereas *Trophoniella hephaistos* has particles on both its dorsal and ventral areas.

The phylogenetic analysis showed *Trophoniella* to be the closest relative of *Piromis* in Flabelligeridae supported by a high bootstrap value (See Fig. 6). Our findings are consistent with previous morphological studies that indicated a close relationship between *Trophoniella* and *Piromis* based on their shared characters such as tongue-shaped lobe, multiarticulated notochaeta, and thick tunic (Salazar-Vallejo 2011b; Salazar-Vallejo et al. 2008).

### Key to species of the genus of *Trophoniella*

The key by Salazar-Vallejo (2012b) is amended with the addition of this new species at couplet 20.

**Table:** 

19	Anchylosed neurohooks from chaetiger 14; neurohooks with accessory tooth longer than fang, eyes absent	***Trophoniella indica* (Fauvel, 1928)**
–	Anchylosed neurohooks from chaetiger 17, or from posterior chaetigers; neurohooks with accessory tooth about as long as fang, eyes present	**20**
20	Anchylosed neurohooks from chaetiger 17–20; Branchial plate tongue -shaped	***Trophoniella hephaistos* sp. n.**
–	Anchylosed neurohooks from chaetiger 40; Branchial plate not tongue-shaped	***Trophoniella enigmatica* Salazar-Vallejo, 2012**

## Supplementary Material

XML Treatment for Trophoniella hephaistos
